# Global evolution and paleogeographic distribution of mid-Cretaceous orbitolinids

**DOI:** 10.14324/111.444/ucloe.000001

**Published:** 2019-08-02

**Authors:** Marcelle BouDagher-Fadel, Geoffrey David Price

**Affiliations:** 1 Office of the Vice-Provost (Research), University College London, 2 Taviton Street, London WC1H 0BT, UK

**Keywords:** Foraminifera, orbitolinids, mid-cretaceous, biostratigraphy, phylogeny, palaeogeographic distribution, extinctions, global anoxic events, sea-level changes, palaeoenvironment, climate, ecology

## Abstract

Members of the Larger Benthic Foraminiferal (LBF) family Orbitolinidae occurred from the Cretaceous to the Paleogene, however, they were most diverse during the mid-Cretaceous, and dominated the agglutinated LBF assemblages described from limestones of that period. Various orbitolinid species have been used to zone and date lithologies formed in the shallow, warm waters of the Aptian to the early Cenomanian, and many, sometimes inaccurate, generic and sub-generic nomenclatures have been proposed to differentiate the often-subtle morphological changes that orbitolinids exhibit over time. Also, until now, it has not been possible to develop an effective global overview of their evolution and environmental development because descriptions of specimens from Asia have been relatively rare. Following our recent study of over 1800 orbitolinid-rich thin sections of material from 13 outcrops of Langshan limestone, from the Southern Tibetan Plateau, and from the Barito Basin, South Kalimantan, Indonesia, it has been possible to compare the stratigraphic ranges of these orbitolinids with previously described Tethyan and American forms, based on the use of a planktonic zonal (PZ) scheme, itself tied to the most recent chronostratigraphic scale. This has allowed the reconstruction of the phylogenetic and paleogeographic evolution of the orbitolinids from their Valanginian origin in the Tethys. Although the Tethys remained the paleogeographic centre for the orbitolinids, it is inferred here for the first time that a bi-directional paleogeographic migration of some orbitolinid genera occurred from the Tethys to the Americas and also to the Western Pacific region. Our observations and dating suggest that global marine regressions in the Aptian were coincident with, and may well have facilitated, these orbitolinid transoceanic migrations. Migration stopped however after rising sea level in the early Albian appears to have again isolated these provinces from each other. Tectonic forces associated with the subduction of the Farallon Plate and further sea level raises led to the opening of the Western Interior Seaway in North America, which correlates with, and may have been the cause of, the middle Albian (top of PZ Albian 2) extinction of the American orbitolinids. The extinction of the orbitolinids revealed that the Western Pacific province was split into two sub-provinces, with extinction occurring at the end of the early Albian (top of PZ Albian 1) in the Northwest Pacific sub-province, and at the end of the Albian (top of PZ Albian 4) in the subprovince that is today South East Asia (on the margins and west of the Wallace Line). The final near extinction of the orbitolinids occurred at the end of the Cenomanian in the Tethyan province, which coincides with, and may have been caused by, global anoxic oceanic events that correlate with a near-peak Mesozoic eustatic sea level high-stand that led to the overall global collapse of the paleotropical reef ecosystem at that time.

## Introduction

The Orbitolinidae are an agglutinated, and now extinct, family of the Larger Benthic Foraminifera (LBF). Orbitolinidae were present in the warm, shallow marine waters of the Early Cretaceous to the early Oligocene, however, they were most diverse during the mid-Cretaceous. During the Early to mid-Cretaceous (Valanginian to early Cenomanian), there was an identifiable increase in the complexity of their morphological structure, which enabled them to house within their tests symbiotic algae [1], and it is these forms which are the subject of this paper. Traditionally, orbitolinids are considered to define two major, distinct paleogeographic realms, namely those of the Americas and the Tethys (see BouDagher-Fadel [1]), but in this study we document forms from the Western Pacific that are distinct from their Tethyan forebears, and so define a third orbitolinid province.

**Figure 1:**
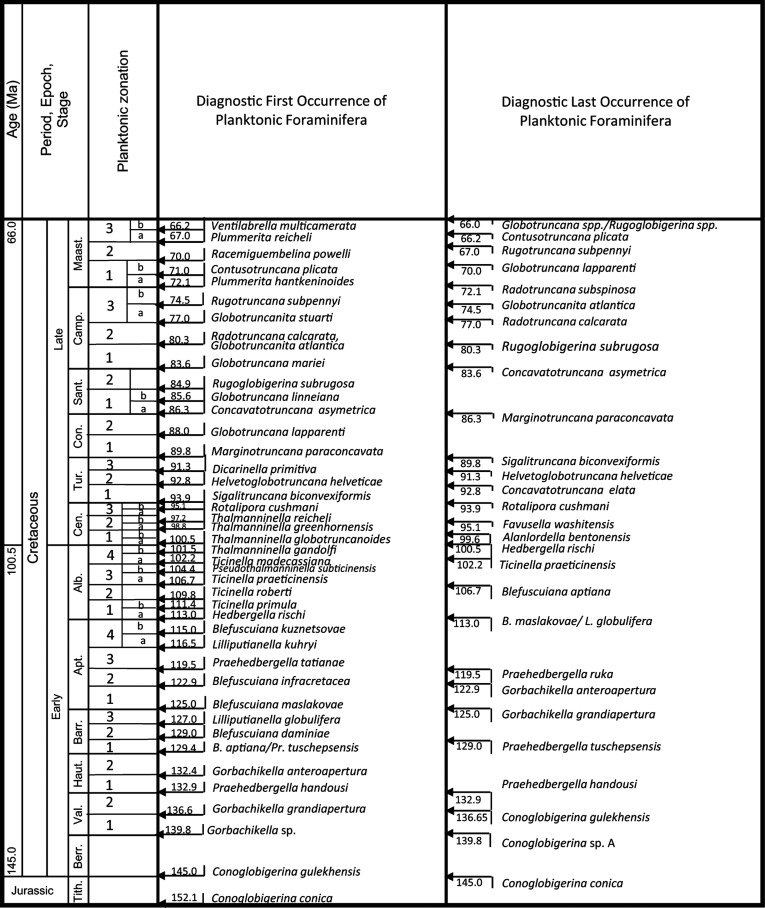
The diagnostic first and last occurrences of Cretaceous planktonic foraminiferal species, calibrated against the most recent biostratigraphic time scale and radio-isotope data (after BouDagher-Fadel [2]).

The symbiotic orbitolinids were rock-forming organisms, and they are found in association with other marine forms, including planktonic foraminifera. This coexistence with planktonic forms, enables their stratigraphic ranges to be defined very precisely, as they can be tied to the high resolution planktonic zonal (PZ) dating scheme of BouDagher-Fadel ([2]; see Fig. 1), which itself is tied to the absolute time scale of Gradstein et al. [3].

Early to mid-Cretaceous orbitolinids have been described from Tethyan limestones from, for example, the Mediterranean [4], Southwest England [5–10], Spain [11, 12], France [13], Italy [14], Israel, Lebanon and Syria [15], Yemen [16], Oman [17–19], Saudi Arabia [20, 21], the United Arab Emirates [22], Iran ([23–27]; Rahiminejad and Hassani, 2016), Afghanistan [28], and Tibet [29–31]. They are also reported from the Northwest Pacific [32], Japan and Sakhalin [33–44], and in Africa, where they are found in Ethiopia [45], Somalia (e.g. [46–48]) and Tanzania [49].

Furthermore, orbitolinids have been reported from the northwestern Atlantic, off the Flemish Cap, Newfoundland [50, 51], and have been described from the Caribbean and the Americas [52], Mexico [53–55], and Venezuela [56].

During their existence, the structurally complex orbitolinids showed relatively rapid phylogenetic evolution, developing many stratigraphically short-ranged species, which when combined with the PZ scheme (see Fig. 1) act as a very important and precise index fossil group for the shallowmarine environments of the mid-Cretaceous Tethys [1, 6, 31, 57]. As a result, they have been widely adopted as a biostratigraphic tool by industry in the exploration of Middle Eastern and other oil and gas fields.

**Figure 2:**
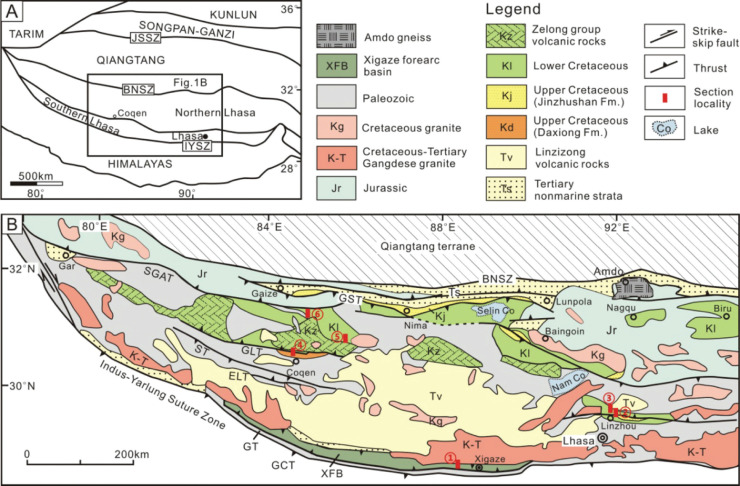
(A) Simplified tectonic map of the Tibetan Plateau and adjacent regions, showing the Lhasa terrane in the context of the Tibetan Plateau [58]. JSSZ, Jinsha suture zone; BNSZ, Bangong-Nujiang suture zone; IYSZ, Indus-Yarlung suture zone. (B) Simplified geological map of the Lhasa terrane modified from [59]. SGAT, Shiquan-Gaize-Amdo thrust; GST, Gaize–Selin Co thrust; GLT, Gugu La thrust; ST, Shibaluo thrust; ELT, Emei La thrust; GT, Gangdese thrust system; GCT, Great Counter thrust. Section 1 from the Xigaze forearc basin; Sections 2 and 3 from the Linzhou basin; Sections 4, 5 and 6 from the Coqen basin.

**Figure 3:**
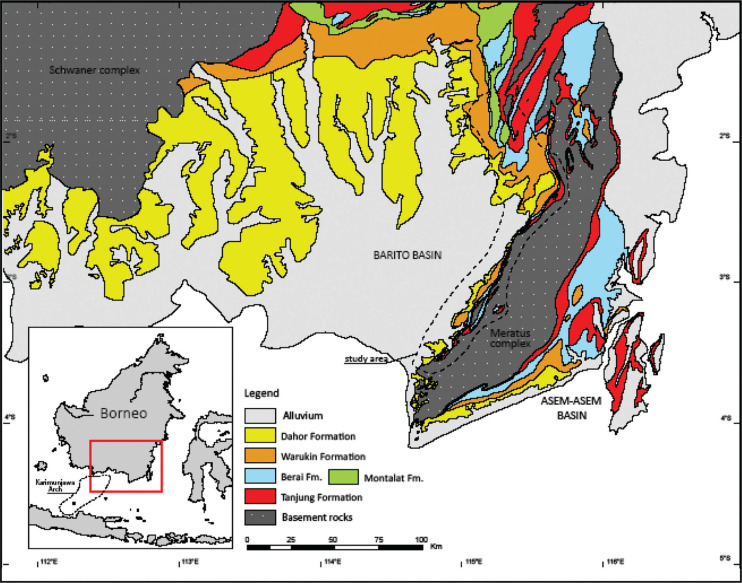
Cenozoic geology of the Barito and Asem-Asem Basins (modified from [61]).

In this paper, the evolution and paleogeographic development of these symbiotic, morphologically complex orbitolinids is inferred from the re-analysis of the published data referred to, and combined with new observations from over 1800 thin sections of material from 13 outcrops of Langshan limestone of the Southern Tibetan Plateau (see Fig. 2), the Sangzugang Formation in Southern Lhasa subterrane (see [60]), the Xiagezi-II section of the Langshan Formation in southern part of the Northern Lhasa subterrane (see [59, 62]), the Azhang and Guolong sections from the Langshan Formation in the Northern Lhasa subterrane (see [31]), the Jingshughan, Langshan, Xiongba, Xiongmei, Baoji, Daya, Gegi, Letie and Zulong sections [63], and the Jiarong and Laxue sections from the Linzhou Basin (see [31]). In addition, material has been studied from the western flank of the Meratus Mountains, an uplifted accretionary collision complex that records suturing of East Java–West Sulawesi to the Sundaland margin during the mid-Cretaceous (see Fig. 3). The uplifted complex now forms the eastern boundary of the Barito Basin, South Kalimantan, Indonesia (see [60]).

**Figure 4:**
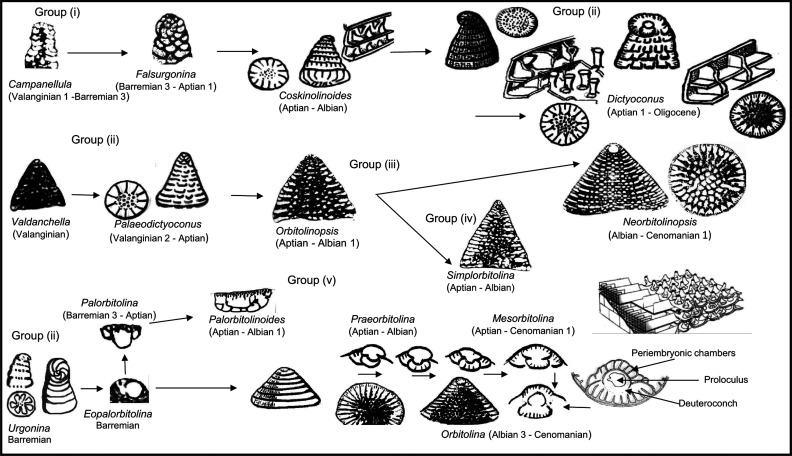
Gradual morphological changes from primitive orbitolinids to the advanced *Orbitolina* in Tethys.

**Figure 5:**
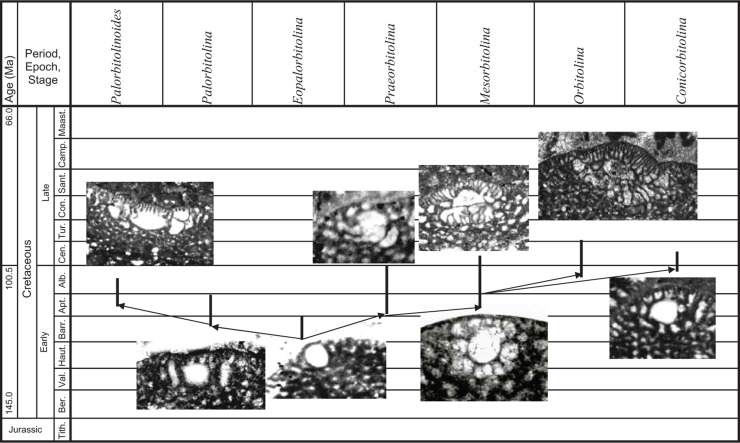
Example of evolutionary Tethyan lineages from morphological Group (ii) to (v).

**Figure 6:**
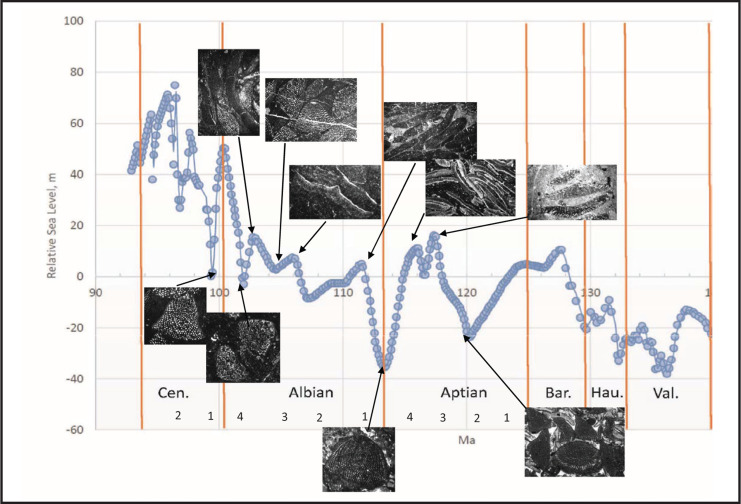
Variation in sea-level during the mid-Cretaceous based on Miller et al. [60] correlated to the boundaries of the PZ after BouDagher-Fadel [2] and showing dominant assemblages at the top of regression and transgression phases.

**Figure 7:**
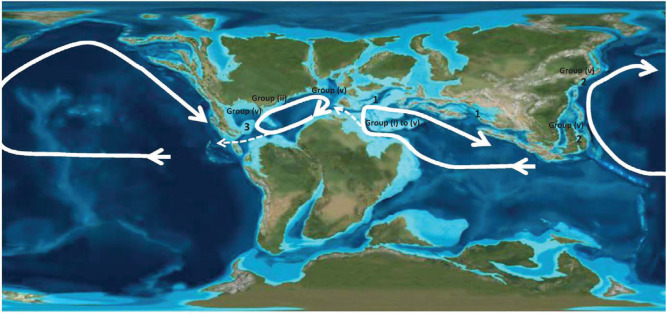
The provincial distribution of the orbitolinids during the Early Cretaceous, Early Albian in the Tethys (1), the Western Pacific (2), and the Americas (3), with paleo-oceanic currents shown by the white arrows.

By correlating these observations and the literature data with our high-resolution PZ scheme ([2], Fig. 1), we are able to infer, for the first time, a comprehensive, global synthesis of the biostratigraphic, phylogenetic, and paleogeographic evolution of these orbitolinids. We infer that the earliest morphologically complex orbitolinids evolved in the Tethys from primitive Valanginian forms such as *Valdanchella, Paleodictyoconus* and *Campanellula* (Fig. 4). More complex forms developed rapidly into different Tethyan phylogenetic lineages (e.g. Figs 4 and 5). It appears that major, global sea level regressions starting in the early Aptian (PZ Aptian 1, 125.0 Ma) and in the late Aptian (PZ Aptian 4, 116.5 Ma; see Fig. 6), correlate with and probably facilitated bidirectional transoceanic migration of orbitolinids. One migration was from the Tethys to the previously recognised American province, but a second migration was from Tethys to the newly defined Western Pacific province (see Fig. 7). These migrations stopped after rising sea level during the early Albian (PZ Albian 1) appears to have isolated the provinces one from another.

The isolated orbitolinids of the Northwest sub-province of the Western Pacific (present day Japan) became extinct at end of the early Albian (top of PZ Albian 1), whereas those in the isolated American province became extinct at the end of PZ Albian 2 (106.7 Ma). All forms in the subprovince that is today South East Asia (on the margins and to the west of the Wallace Line) went extinct at the end of the Albian (top of PZ Albian 4). The ‘hotspot’ for orbitolinid evolution, however, remained in Tethys, where environmental conditions continued to contribute to their success until the end of the Cenomanian, when virtually all symbiotic, morphologically complex orbitolinids became extinct, as indeed did many of the other agglutinated LBFs that dated from the Early Cretaceous and Jurassic (see [1]). These extinctions coincided with an anoxic oceanic event [64], and correlate with a near-peak Mesozoic eustatic sea level high-stand (see Fig. 6, and [65]).

## Morphological characteristics of orbitolinids 

**Figure 8:**
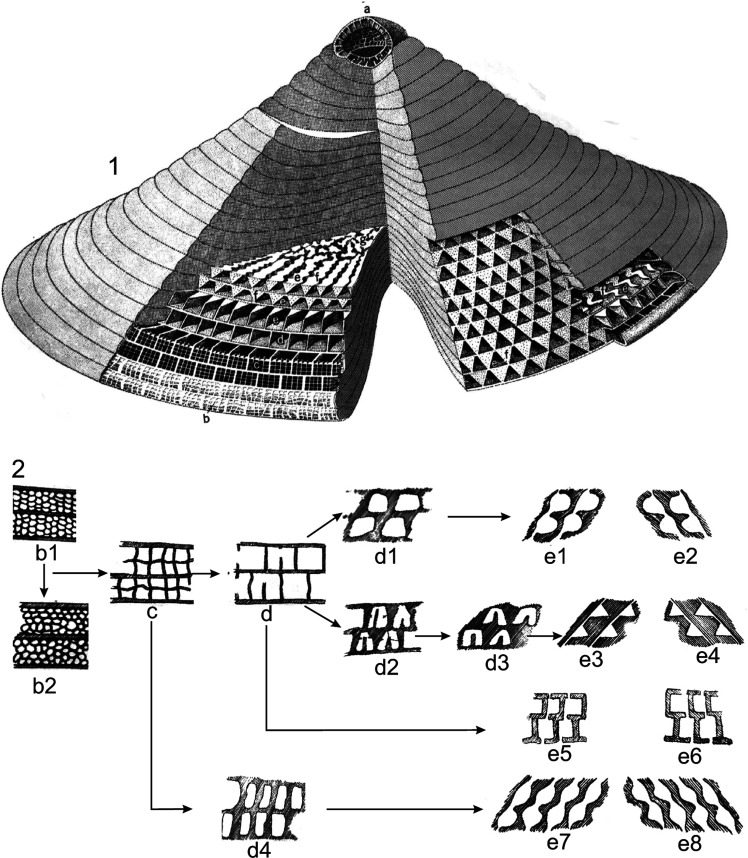
The test architecture of *Orbitolina* (not to scale). (1) Test dissected in several places to show the internal structures (after Douglass [52]); (2) Diagrams showing micro-structures of *Orbitolina* exposed by tangential sections cut progressively deeper below the epidermis: a, Megalospheric embryonic apparatus; b, slightly eroded surface exposing sub-epidermal cells; b1–b2, regular/irregular arrangement of secondary epidermal cells (stage III); c, primary sub-epidermal cells (stage II); d, marginal chamberlets (stage I) with residual traces of vertical primary subepidermal plates only; d1–d4, sections through marginal chamberlets between (c and d) and the beginning of the true radial chamber-passages with canals (e1, e3, e5, e7). e1–e2, Radial chamber passages sub-rounded, canals short, wall thickness relatively small; e3–e4, radial chamber passages triangular, canals long, wall thickness relatively great; e5–e6, radial chamber passages initially rectangular, with simple perforations, wall thickness small; e7–e8, radial chamber passages irregular – rounded originating from vertical pairs of primary sub-epidermal cells, canals short, wall thickness small; f, main triangular partitions with a zigzag shape when seen deeper in the test; g, the complex central zone.

The orbitolinids are members of the order Textulariida, which have agglutinated tests that are made of foreign particles bound by organic/calcitic cement. They are characterised by having conical tests, subdivided into numerous chambers, and are usually a few millimetres in height and diameter (although as noted, some forms attained diameters of 5 cm or more). The numerous uniserial discoidal chambers are partially subdivided by radial or transverse partitions, or pillars. They have cribrate, areal apertures (see Fig. 8).

**Figure 9:**
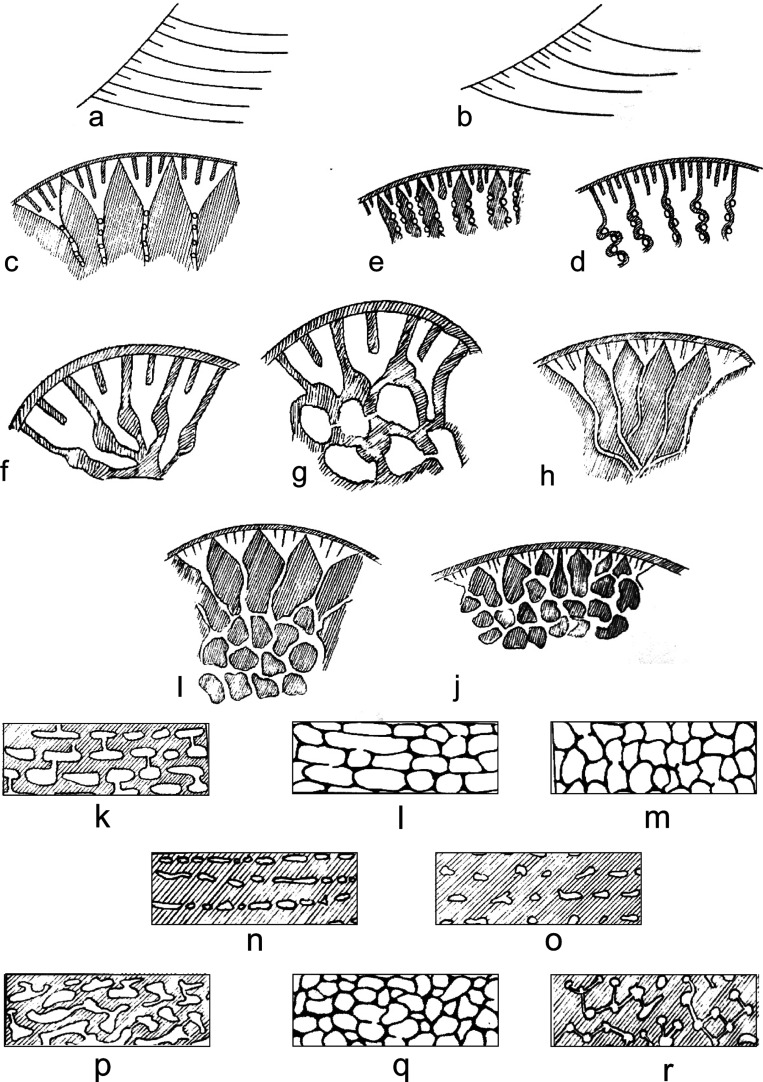
(a–b) Diagrammatic axial sections of orbitolinids; a, showing closely spaced chamber layers with primary horizontal sub-epidermal plates only; b, showing widely-spaced chamber layers with primary and secondary horizontal sub-epidermal plates. (c–e) Diagrammatic basal sections of orbitolinids; c, marginal zone broad, marginal chamberlets triangular, radial walls thick, straight; d, marginal zone broad, marginal chamberlets rectangular, radial walls thin, zigzag; e, marginal zone narrow, marginal chamberlets sub-triangular, radial walls moderately thick, vertical primary sub-epidermal plates thickening inward with some prolonged as radial walls. Note: In species having triangular radial passages, the thickness of radial walls as seen in basal views will vary according to the position on the section just above or just below a chamber floor. (f–j) Diagrams illustrating radial and reticular zones of orbitolinids as seen in basal views; f, radial partitions; g, reticular partitions; h, radial chamber passages; i, radial and reticular chamber passages; j, complex reticular zone, no radial zone. (k–r) Diagrams showing various textures of the central zone in *Orbitolina* as observed in axial and oblique sections; shaded areas and lines represent shell material. (k–o) Axial sections; k, wall and floor thickness and chamber diameters sub-equal; chamber layers clearly marked and connected by short, sub-vertical canals; l, wall and floors thin; chamber layers clearly connected by simple perforations and clearly recognisable by alignment of longitudinal segments of chamber passages in the radial zone; m, wall and floors thin; chamber layers clearly connected by simple perforations but not clearly recognisable; the section is cut through the reticular zone and chamber segments are all more or less transverse; n, wall thickness small, floor thickness relatively great; chamber layers clearly marked and connected by oblique canals not visible in axial sections; o, wall and floor thickness great; chamber layers not clearly recognizable owing to wide spacing of chamber segments; oblique canals not visible in axial sections. (p–r) oblique sections; p, oblique section corresponding to (a) above; labyrinthic texture; canals (when visible) not clearly differentiated from chamber segments; q, oblique section corresponding to (c) above; that corresponding to (b) would be similar but would show a few longitudinal chamber segments; cellular texture; r, oblique section corresponding to (d) or (e) above; dentritic texture; small chamber segments connected by long, oblique canals forming a roughly polygonal network.

**Figure 10:**
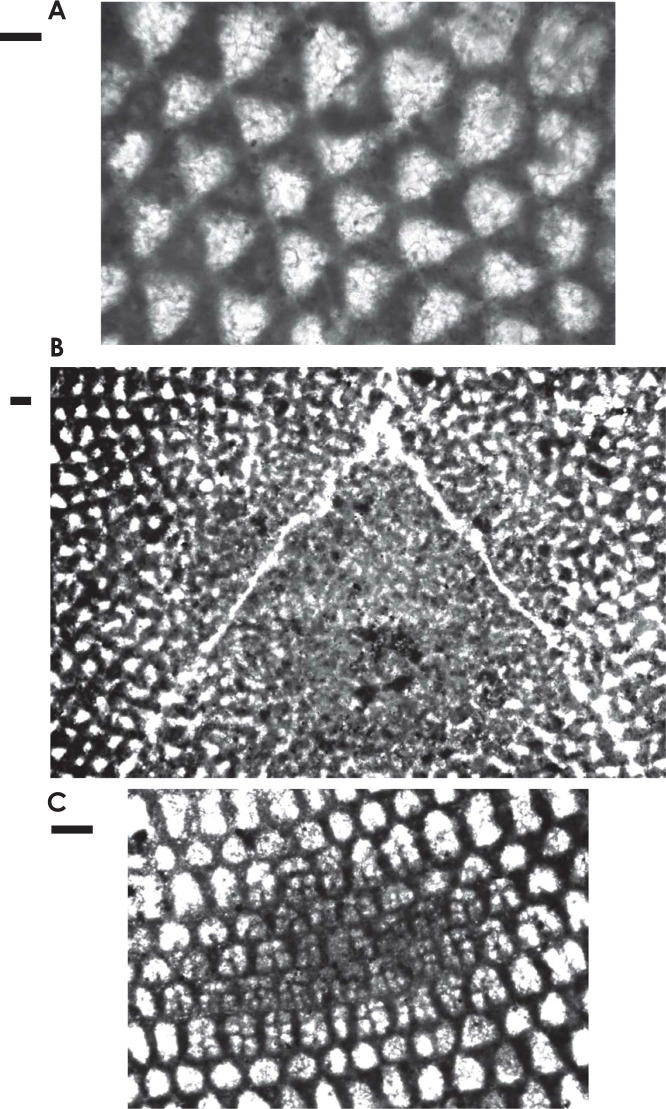
Enlargement of parts of *Palorbitolina lenticularis* (Blumenbach) figured by BouDagher-Fadel [1], scale bar = 100 μm. (A) Transverse section showing the triangular main partitions; (B) the same transverse section showing the central complex reticular part of the test; (C) the same transverse section showing the subdivision of the marginal chamberlets into cellules/chamberlets.

**Plate 1:**
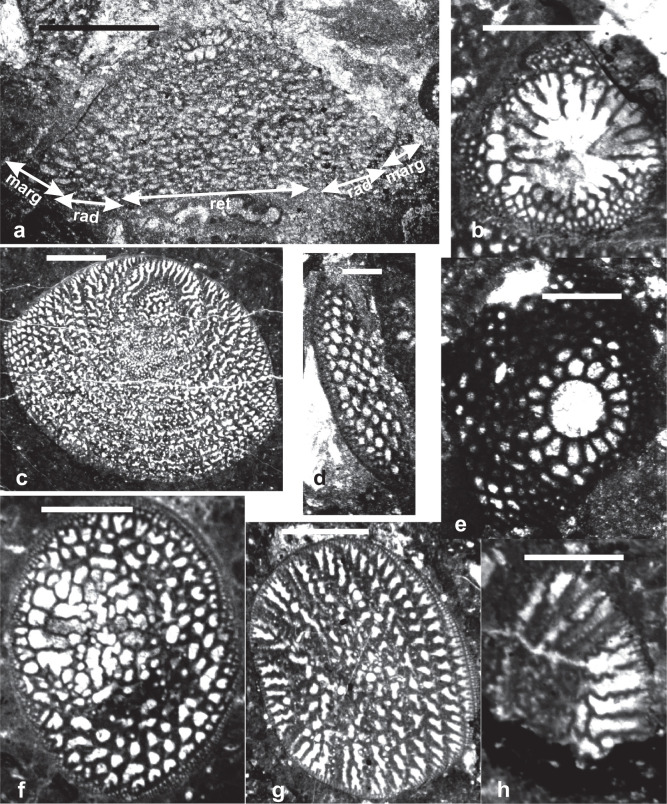
Scale bars: a, c = 1 mm; b, d–h = 0.5 mm. Key words: marg = marginal zone; rad = radial zone; ret = reticular zone. All samples are deposited in School of Earth Sciences and Engineering Nanjing University. a–c, f. *Mesorbitolina aperta* (Erman), Langsham Formation, Tibet, PZ Albian 3–Cenomanian 1: a) Axial section through the megalospheric embryonic apparatus; b) basal section of the megalospheric embryonic apparatus; c) thin section showing the details of the radial zone; f) thin section through the reticular zone. d. *Mesorbitolina* sp., Tibet, Aptian. Thin section through the marginal zone. e. *Mesorbitolina subconcava* (Leymerie), Indonesia, PZ Albian 1. Basal section through embryonic apparatus showing the periembryonic chambers. g–h. *Mesorbitolina texana* (Roemer), Tibet, PZ Aptian 4. Random thin sections: g) showing details of the radial and reticular zones; h) basal view showing the zigzag main partitions with apertural pores at the reentrants. The partitions are broken up in the central complex reticular zone.

The Cretaceous morphologically complex orbitolinids are divided into the dictyoconines and orbitolinines, and range from the Valanginian to the Cenomanian. They are divided into the following five morphological groups (see [1]):

- Orbitolinids with no complex central zones (e.g. *Campanellula*, PZ Valanginian 1). They lack thick radial partitions and pillars in the central zone.
- Orbitolinids with a complex central zone and radial partitions thickening away from the periphery and breaking up into pillars in the central zone, first appeared in the late Valanginian with developed peripheral tiered rectangular chamberlets. They evolved into the dictyoconines (e.g. *Paleodictyoconus*, PZ Valanginian 2, Fig. 4; *Paracoskinolina,* PZ Barremian 1–Albian 4), or into the orbitolinines (e.g. *Urgonina,* PZ Barremian 1, Fig. 4) from forms with the outer parts of their chambers lacking partitions but with interseptal pillars connecting the adjacent septa.
- Orbitolinids with radial partitions thickening away from the periphery to anastomose centrally around the aperture and form a reticulate zone in the transverse section, also first appeared in the late Valanginian (e.g. *Valdanchella,* PZ Valanginian 2). The peripheral zones of their chambers are subdivided into rectangular chamberlets by fine radial partitions (Fig. 4).
- Orbitolinids with radial partitions that became zigzagged, thickening and fusing centrally, giving a stellate appearance in the transverse section, first appeared in the Aptian (e.g. *Simplorbitolina,* PZ Aptian 1-Albian 4). Their tests may have tiered peripheral chamberlets (e.g. *Dictyoconella,* PZ Cenomanian 3-Maastrichtian 3, Fig. 4).
- Orbitolinids with radial partitions thickening, with triangular cross-sections away from the periphery and anastomosing in the central area, first appeared in the Barremian (e.g. *Eopalorbitolina*, PZ Barremian 1, Fig. 5) and evolved rapidly in the mid-Cretaceous. The test of these orbitolinids is defined by the shape of the embryonic apparatus, and by the size and shape of the chamber passages that can be seen in tangential sections. The earliest formed chambers of the megalospheric generation can form a complex embryonic apparatus, which can be divided into a protoconch, a deuteroconch, a sub-embryonic zone and peri-embryonic chamberlets (see Plate 1, b, e; Fig. 4). In the axial section, the embryo is located at the apex of the cone, followed by a series of discoidal chamber layers. The embryonic apparatus evolved from a simple apparatus, consisting of a large globular fused protoconch and deuteroconch, followed by peri-embryonic chambers as in *Palorbitolina*, to an embryonic apparatus divided into a protoconch and deuteroconch but a not completely divided sub-embryonic zone, as in *Praeorbitolina*. This latter evolved in turn into forms in which the deuteroconch and sub-embryonic zone are more or less of equal size, as in *Mesorbitolina *(Plate 1, d). In *Conicorbitolina* (Plate 3, f) the marginal zone became extensively divided by vertical and horizontal partitions, while in *Orbitolina* the deuteroconch is highly subdivided and of much greater thickness than the sub-embryonic zone (see Figs 4, 5; [6, 28, 31, 57, 66, 67]). In transverse section, the chambers are seen divided into a marginal zone, with sub-epidermal partitions, and a central zone with radial partitions (Plate 1, a; Figs 8.1–8.2, 9). The chamber passages are formed in the radial part of the central zone of each chamber layer (Figs 8.1–8.2, 9h), where each chamber passage is subdivided by vertical main partitions, which are prolongations of the vertical main partitions of the marginal zone (Figs 9a–f, h). The radial partitions (Fig. 9f) in advanced orbitolinids (e.g. *Mesorbitolina, Orbitolina*) thicken away from the periphery and anastomose in the central area, producing an irregular reticular network (Plate 1, c, f–h; Figs 9g, i–j; Fig. 10b). In cross-section, the chamber passages can be triangular (Figs 9c, 10a), rectangular (Fig. 10c) or oval, or can show a gradation between shapes (Fig. 9e) [28]. In the radial zone of *Orbitolina,* the stolons are arranged in radial rows alternating from one chamber to the next one (see [1]). Their alternating position would have obliged the protoplasm to flow in an oblique direction [66]. In the annular radial zone of the conical test (Plate 1, a, c, g), radial septula subdivide the chambers into radial compartments with various thickness and textures (Plate 1, h; Fig. 9f, h, k–r), narrowing towards the centre to fuse into a reticular network (Plate 1, a, f, h; Fig. 9g, i, j) which minimizes the volume of chamberlet cavities (Plate 1, a, f–h).

## Biostratigraphy, phylogeny and paleogeographic distribution of the orbitolinids

**Plate 2:**
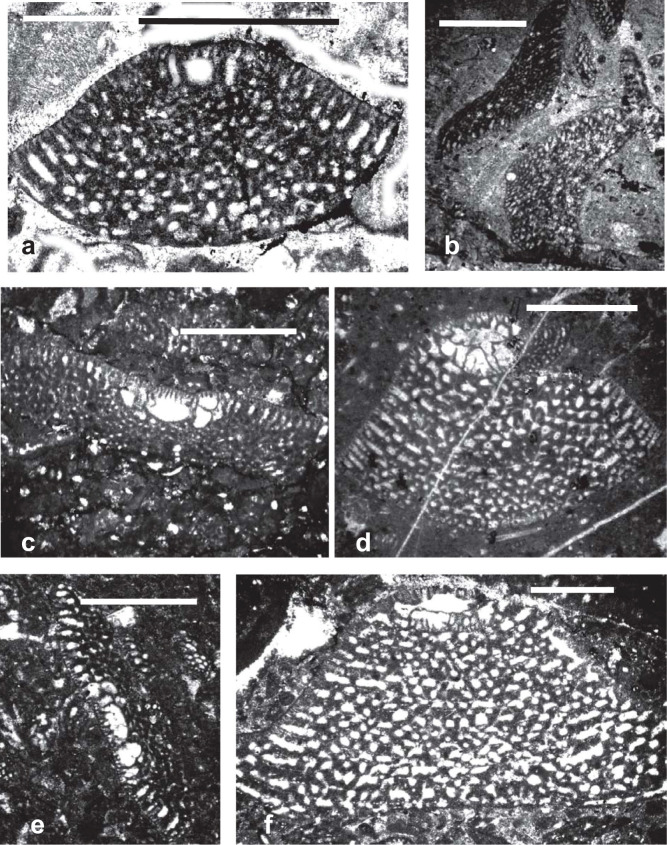
Scale bars = 1 mm. All samples are deposited in School of Earth Sciences and Engineering Nanjing University. a. *Palorbitolina lenticularis* (Blumenbach), Jiarong section, TLK1a, PZ Aptian 2, sample 14LZ13. b. *Praeorbitolina cormyi* Schroeder, Laxue section, TLK1a, PZ Aptian 2, 14 LZ12 c. *Palorbitolinoides orbiculatus* Zhang, Langsham section, TLK1a, PZ Aptian 2, 16SL 02. d. *Mesorbitolina aperta* (Erman). Guolong section, TLK1h, PZ Cenomanian 1, 13GL33. e. *Palorbitolinoides hedini* Cherchi and Schroeder, Langsham section, TLK1e, PZ Albian 2, 16SL45. f. *Mesorbitolina subconcava* (Leymerie), Langsham section, TLK1c, PZ Aptian 4b, 16SL29.

**Plate 3:**
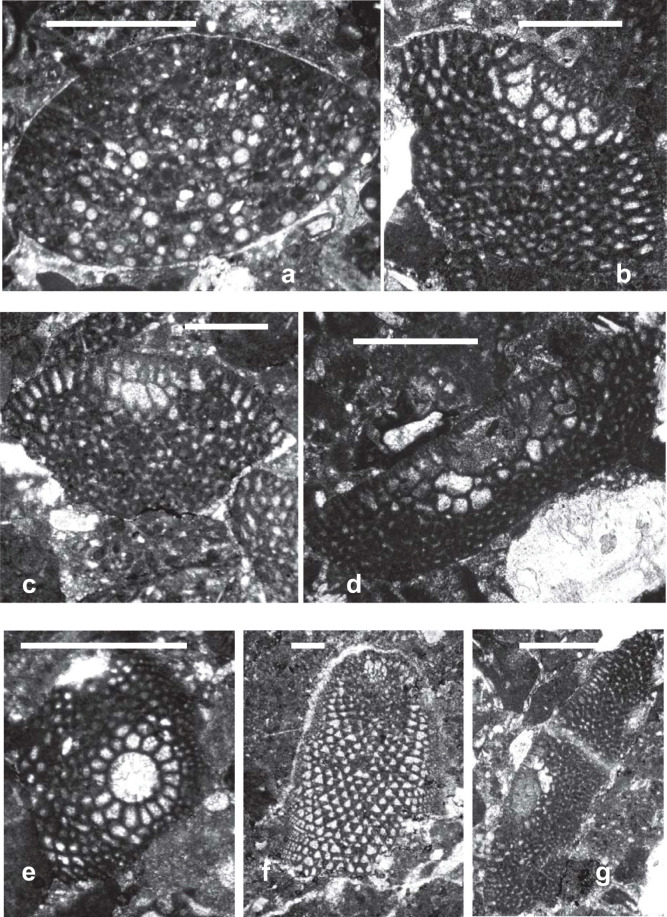
Scale bars = 1 mm. All photos are from sections from the western flank of the Meratus Mountains, Barito Basin, Southeast Kalimantan, Indonesia. All samples are deposited in UCL Collections. a–c. *Mesorbitolina texana* (Roemer), PZ Albian 1, BBr-14. 2–3) vertical sections. d, g. *Palorbitolinoides orbiculatus* Zhang, PZ Albian 1, BBr-14. e. *Mesorbitolina subconcava* (Leymerie), PZ Albian 1, BBr-14, oblique transverse section through embryonic apparatus. f. *Conicorbitolina* sp., PZ Albian 4, BBr-22.

The orbitolinids are very useful biostratigraphic markers in early to mid-Cretaceous Tethyan carbonate, siliciclastic or mixed deposits [28, 31, 68]. They have short ranges and are, with practice, easily identified in thin sections (e.g. see Plates 2 and 3). Orbitolinids show provincialism unlike some LBFs of the period (e.g. the miliolides). Traditionally, they have been considered to define two major, distinct paleogeographic realms, namely those of the Americas and the Tethys (see [1]).

Many forms from the morphological Group (i) described evolved gradually to more advanced forms of Groups (ii) to Groups (vi). Notable and characteristic paraphyletic lineages include:

- *Campanellula–Paracoskinolina–Coskinolinoides–Dictyoconus* (PZ Valanginian 1–Cenomanian 3; all forms in this group became extinct in the Cenomanian, except *Dictyoconus,* which persisted to the Oligocene). The evolutionary trend of this Group (i)–Group (ii) lineage is characterised by an increase in test diameter and the development of increasingly complex radial partitions radial partitions thickening away from the periphery, that break up into pillars in the central zone, forming highly developed and complex layers of chamberlets.
- *Valdanchella–Paleodictyoconus–Montseciella–Rectodictyoconus–Simplorbitolina–Neorbitolinopsis* (PZ Valanginian 1–Cenomanian 1). The evolutionary trend of this Group (ii)–Group (v) lineage is characterised by the increase in size, a gradual enlargement of the whole embryo the development of the megalospheric embryo in a centric or near centric position, and the development of increasingly complex radial partitions, becoming zigzagged with a stellate appearance in the transverse section (as in *Simplorbitolina*) or thickened and fused centrally forming highly developed and complex layers of chamberlets (as in *Neorbitolinopsis*).
- *Urgonina – Eopalorbitolina - Palorbitolina – Palorbitolinoides* (PZ Barremian 1–Albian 1). The evolutionary trend of this Group (ii)–Group (v) lineage (see Fig. 5) is related to the formation of peripheral, tiered, rectangular chamberlets in two or more series, the shape and position of the embryonic apparatus from a bi-chambered embryo in a clear eccentric position, with a missing peri-embryonic zone in *Eopalorbitolina* (see Fig. 5), to the development and the increase in size of the peri-embryonic zone to embrace more and more of the embryonic apparatus, surrounding the upper half of the nearly centric embryonic chamber in *E. transiens*, and becoming completely annular surrounding the upper part of the centric embryonic chamber in *Palorbitolina lenticularis* (Figs 5, 10). In *Palorbitolinoides* (e.g. *Palorbitolinoides hedini* Cherchi and Schroeder [69], Plate 2, e) the large and flattened embryonic chamber is surrounded by a developed inflated peri-embryonic zone.
- *Praeorbitolina–Mesorbitolina–Orbitolina–Conicorbitolina* (PZ Aptian 1–Cenomanian 3). The main evolutionary characters in this Group (v) lineage are the position of the embryonic apparatus, which is in an eccentric position in earlier forms (e.g. *Praeorbitolina*), but centrally placed in advanced forms, consisting of the protoconch and the deuteroconch (e.g. *Mesorbitolina*). In *Conicorbitolina* (PZ Albian 4–Cenomanian 1) the large proloculus is divided into a protoconch and deuteroconch, with the marginal zone becoming extensively divided by vertical and horizontal partitions (Plate 3, f; Fig. 5). The main evolutionary characters of this *Orbitolina* (PZ Albian 3–Cenomanian 3) is the increase in size of the apically situated embryonic apparatus, where the deuteroconch becomes about 3 times thicker than the sub-embryonic zone (Figs 5, 9).

On the basis of this study, and using the lineages described, we are able to establish for the first time that there were in fact three distinct paleogeographic provinces for these symbiotic, morphologically complex orbitolinids (see Fig. 7); namely the previously defined American province (including current day Texas, Venezuela, Mexico), a Tethyan province (including Europe and the Flemish Cap off Newfoundland, Arabia, Turkey, Iran, Lebanon, Oman, Syria, Qatar, Tibet), and a newly identified Western Pacific province, which is divided into two sub-provinces; the subprovince of Northwest Pacific, which includes Japan and the Philippine island of Cebu, and a subprovince that includes what is today South East Asia (west of the Wallace Line).

In Tethys, morphologically complex orbitolinids and their precursors are common from the Valanginian (PZ Valanginian 1) to the Cenomanian (PZ Cenomanian 3), and exhibit several of the phylogenetic lineages described, while in the Americas orbitolinids are only found between the early Aptian (PZ Aptian 1) and middle Albian (PZ Albian 2), and are predominantly represented by the Group (v) genera *Palorbitolina* and *Mesorbitolina*. In the Western Pacific, unidentified and unconfirmed orbitolinids have been listed in the literature as dating from the late Hauterivian to the early Aptian (see [41]). These early forms are however contested, but the Group (v) *Praeorbitolina–Mesorbitolina* lineage is definitely confirmed from PZ Aptian 1 to PZ Albian 1 in Northwest Pacific sub-province and to PZ Albian 4 in the South East Asia sub-province.

**Figure 11:**
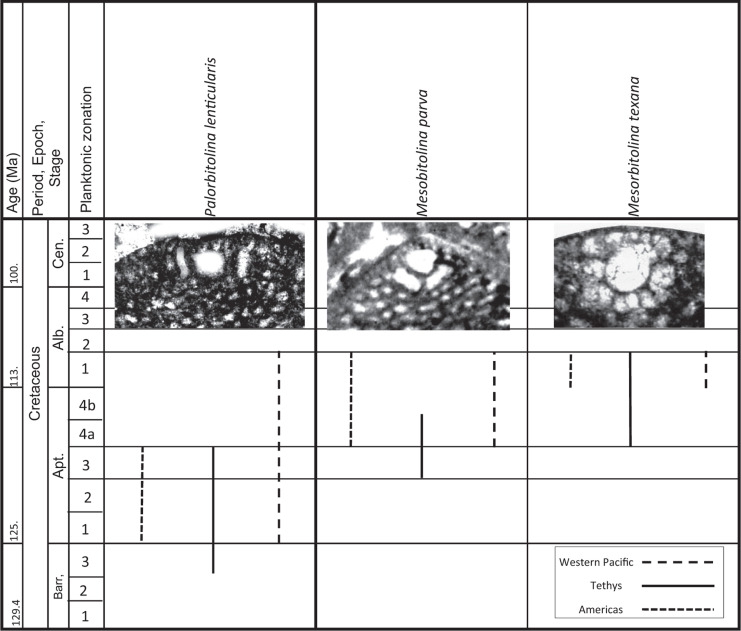
Range chart for some key orbitolinid species from Group (v) in the Tethyan and Western Pacific, and the American provinces.

From this global pattern, we infer that the original hotspot for the evolution of the complex orbitolinids was Tethys, but as will be described, following migration events in the early Aptian out of Tethys, some lineages of the orbitolinids spread to the other provinces. It seems that the migration stopped after the early Albian, and that the provinces were again isolated. There then developed provincial, parallel, but specifically distinct evolutionary trends, until the subsequent provincial extinctions in the Americas and the West Pacific (see Fig. 11).

### The Tethys

Throughout the Tethyan province orbitolinids of Groups (i–v) evolved many lineages. They became morphologically complex and widespread, and are often associated with calcareous algae. Their main Tethyan lineages which evolved from Group (i) include:

- Group (ii)–Group (iv) *Valdanchella–Paleodictyoconus–Montseciella–Rectodictyoconus–Simplorbitolina–Orbitolinopsis* (PZ Valanginian 1–Cenomanian 1);
- Group (v): *Eopalorbitolina–Palorbitolina–Palorbitolinoides* (PZ Barremian 1–Albian 1);
- Group (v): *Praeorbitolina–Conicorbitolina* (PZ Aptian 1–Cenomanian 1).

The orbitolinid assemblages of Western Tethys, the southern Neo-Tethys margin and of southwest Europe are similar to those of the Tibetan carbonate platforms, and they all form a part of the Tethyan realm [31]. All cosmopolitan orbitolinids appeared in the Tethys before spreading to other provinces. For example, in Tethys, *P. lenticularis* (Plate 2, a; Fig. 10) first occurred in late Barremian (PZ Barremian 3, 127 Ma; [31]), 2 million years before its first appearance in the American and Western Pacific provinces at the beginning of the Aptian (PZ Aptian 1, 125.0 Ma). The oldest *P. lenticularis* recorded in what is today the ‘American’ continent was recorded by Schroeder and Cherchi [51] from the late Barremian of the Flemish Cap, North West Atlantic. From the palaeogeography of the time, however, we infer that at this stage the Flemish Cap was the extreme extension of the north western Tethyan realm and was isolated from the more southerly parts of the American province (see Fig. 7).

The earliest *Mesorbitolina* (e.g. *Mesorbitolina lotzei*), likewise, appeared first in Tethys, in PZ Aptian 2. The cosmopolitan *Mesorbitolina parva–Mesorbitolina texana* first appearing in the late Aptian (PZ Aptian 3, 119.5 Ma), 3 million years earlier than in the American and Western Pacific provinces where their first appearances are recorded in PZ Aptian 4, 116.5 Ma [6, 31, 32, 52]. Subsequent to its first appearance, the Tethyan *Mesorbitolina* evolved many phylogenetic lineages, which show the typical evolution from having a simple embryonic apparatus to developing a more complicated one. The most common late Aptian–Cenomanian (PZ Aptian 4–Cenomanian 1) lineage being the *M. texana–M. aperta* lineage (see Plate 1, a–c, 6; Plate 2, d; Fig. 5), where the open deuteroconch in the square embryonic apparatus evolves into a deuteroconch subdivided in the upper part by several partitions of different sizes, whereas the lower part exhibits an irregular network of partitions [31]. No equivalent lineage is found in the other provinces, suggesting that by this stage the provinces were again isolated one from another.

All the main Tethyan orbitolinids became extinct at the end of the Cenomanian, with the exception of rare forms which persist in the Late Cretaceous of the Mediterranean Neo-Tethys, e.g. *Pseudorbitolina, Orbitolinella, Calveziconus, Dictyoconella* and *Dictyoconus* which continues to the Oligocene.

### The Western Pacific

In the Western Pacific province, orbitolinids limestones, associated mainly with Cretaceous arc volcanics, form two sub-provinces. One occurs north along the Eurasian continental margin to the Philippines and Japan, and the other is to the south, along a belt near the Early Cretaceous margin of Sundaland, in what is today South East Asia [36, 70].

In the Northwest Pacific sub-province, reported occurrences of orbitolinids are patchy with numerous doubtful identifications, but those with certain identification belong to Group (v). *Palorbitolina lenticularis* is first recorded from the beginning of PZ Aptian 1 (125.0 Ma, 2 million years after its first appearance in Tethys) in the eastern Philippines (Cebu) and Japan [32, 70].

In the South East Asian sub-province, orbitolinids are more common and occur from PZ Aptian 1 (125.0 Ma) to PZ Albian 4 (100.5 Ma). In West Sarawak and Northwest Kalimantan, orbitolinid-rich beds are recorded from the early Aptian (PZ Aptian 1) of Pedawan and Seberoeang Formations [71]. In North-Central Kalimantan orbitolinids are documented from the Aptian to early Albian (PZ Aptian 1–Albian 1) of the Selangkai Formation in the Upper Kapuas River region [70]. Other Early Cretaceous orbitolinid localities include Southeast Kalimantan along the Meratus Mountains front East of Martapura [72], in South Sumatra, Ratai Bay, Lampung [73], in the Gumai Mountains [74], Central Java [75], and West Sulawesi [76, 77].

The Western Pacific orbitolinids are mainly of Tethyan origin belonging to Group (v). Aptian forms originally described as endemic to the South East Asia sub-province are in fact found to be synonyms to the Tethyan forms. As an example, *Orbitolina scutum* and *Orbitolina trochus* originally named as *Patellina scutum* von Fritsch, 1878 and *Patellina trochus* von Fritsch, 1878*,* are both described from Borneo and were assumed to be of Eocene age by von Fritsch [78], but were later re-identified as the Tethyan species *P. lenticularis* and *M. parva* [72]. While *P. lenticularis* ranges from late Barremian to early Aptian in Tethys, it is only recorded from the Aptian in the Western Pacific.

Sikumbang [79] recorded quoting Rolf Schroeder's identifications of Meratus Range orbitolinids as *P. lenticularis* and *M. parva*, indicating an early late Aptian age. In addition to these forms, we record in this work for the first time the presence of the late Aptian to early Albian (PZ Aptian 3–Albian 1) Tethyan species of *Palorbitolinoides orbiculatus* (Plate 2, c; Plate 3, d, g) in the early Albian (PZ Albian 1) of the western flank of the Meratus Mountains, Barito Basin, Southeast Kalimantan, Indonesia. The Tethyan genus *Conicorbitolina* which evolved from *Mesorbitolina* in Tethys in Albian 3 and ranges to Cenomanian 1 (see Fig. 7) is also recorded here for the first time from the late Albian (PZ Albian 4) of the Barito Basin, Kalimantan*, Conicorbitolina* sp. (Plate 3, f). Although the shape of the test is similar to the Tethyan *Conicorbitolina conica,* those from Southeast Kalimantan vary in the shape and number of periembryonic chambers (see Plate 3, f). This is an example of parallel evolution, which gave rise to a similar but distinct form from that found in the Tethyan province. We infer, therefore, that following their initial migration to the Western Pacific, the *Mesorbitolina* lineage subsequently split into parallel lineages evolving at different rates within the two provinces, apparently with no further gene flow, suggesting that the provinces again became isolated one from another.

The *Praeorbitolina–Mesorbitolina* lineages are represented in the Western Pacific province by *Praeorbitolina cormyi* (Plate 2, b), *Praeorbitolina wienandsi, Mesorbitolina parva*, and *M. texana* (Plate 1, g–h; Plate 3, a–c), and have been recorded from the late Aptian to early Albian (PZ Aptian 3–Albian 1), again 5.5 million of years after their first appearance in Tethys. *Mesorbitolina subconcava* (Plate, 1, e; Plate 2, f; Plate 3, e) is recorded here for the first time from the early Albian (PZ Albian 1) of the Barito Basin, Southeast Kalimantan, 3.5 million of years after its first appearance in Tethys.

Groups (i-iv) forms seem to be missing from the Western Pacific province, unlike in the Tethys. Also, unlike the Tethyan realm, the orbitolinids do not survive the Albian-Cenomanian boundary, but disappeared completely from Japan at the end of PZ Albian 1 [32] and, as shown here, from the Barito Basin, Southeast Kalimantan, Indonesia at the end of PZ Albian 4. No orbitolinids are known from east of the Wallace Line in East Indonesia and Australia-New Guinea regions [80], as these foraminifera required a tropical shallow marine setting, which was not present at this time along the North West Australian margin.

### The Americas

Tethyan orbitolinids belonging to Groups (ii) and (v) seem to have migrated into the American province, however, at a much later date than their first appearance in Tethys.

The American province, unlike the Western Pacific province, contains representatives of the dictyoconines from Group (ii). *Paracoskinolina,* which first appeared in the Barremian (PZ Barremian 1) (or in the late Hauterivian if «*Paracoskinolina*» *praereicheli* Clavel et al. [81], from the late Hauterivian-early Barremian of the Urgonian platform, South East France, Swiss and French Jura, Swiss Prealps, is considered as a *Parakoskinolina*) of Tethys, and *Dictyoconus,* which first appeared in the Aptian (PZ Aptian 1) of Tethys, first appeared in the Albian (PZ Albian 1) of Texas, Mexico, and Venezuela (Maync, 1955 76; Arnaud Vanneau and Sliter, 1995), and range to PZ Albian 2. Species such as *Paracoskinolina sunnilandensis* [82] (PZ Albian 2) and *Dictyoconus walnutensis* (Carsey, 1926) (PZ Albian 1 -2) are unique and indigenous to the American province, and forms recorded as the same as Tethyan species are in fact incorrectly identified. This unique occurrence excludes a West to East migration [83], and confirms that for most of the Albian the American and Tethyan provinces were ecologically isolated one from another.

The earliest form from Group (v) reported from the American province is *P. lenticularis* from PZ Aptian 1 (125.0Ma) in deposits of south Mexico, appearing 2.0 million years later than its first occurrence in the late Barremian of Tethys. The Tethyan *Mesorbitolina* are also widespread in the bank and reef deposits of Texas, New Mexico, Arizona, Guatemala, Honduras and Venezuela (PZ Aptian 4 - Albian 2). The cosmopolitan forms, *M. texana–M. parva* group occurring from PZ Aptian 3–Albian 1 (119.5-109.8 Ma) in Tethys [31], are only reported from the PZ Aptian 4–Albian 2 (116.5-109.8 Ma) of Texas, with *M. parva* only found in the PZ Albian 2 of the Americas.

In the early Albian, species of *Mesorbitolina* continued to thrive in the Americas but developed provincial specific forms, not found in the Tethys or Western Pacific provinces. Thus, the American lineage *Mesorbitolina minuta– Mesorbitolina gracilis–Mesorbitolina crassa* of the PZ Albian 1–2 [52, 84] indicates that once the orbitolinids were established in the American province in the latest Aptian, they evolved independently from, yet in a parallel way to, their Tethyan ancestors, by means of gradual development of their embryonic apparatus. Those American species that had been previously reported from the Tethys or the Western Pacific were in fact misidentified. For example, the American *M. minuta* was reported by Matsumaru and Furusawa [43], from central Hokkaido, but was re-identified as *M. texana* by Cherchi and Schroeder [85].

## Discussion

The Early Cretaceous is believed to have been a greenhouse period, with high atmospheric carbon dioxide concentrations [86], high global average temperatures with sea surface temperatures exceeding 32°C [87, 88], and a stable climate [81]. The earliest Cretaceous (Berriasian–Hauterivian) was also characterised by a sustained period of global low sea levels, which were replaced in the Barremian by a significant global sea level transgression (see Fig. 6), reaching its maximum at around 129 Ma, Barremian 2. This sea level rise flooded low-lying continental regions and so created new ecological niches around the globe, one of which was filled in Tethys by the evolving orbitolinids.

The globally warm period continued in the mid-Cretaceous and was characterised by an increase in the number of agglutinated foraminiferal forms having large alveoles, such as the lituolid *Pseudocyclammina*, or forms with internal radial partitions, such as the orbitolinids (see [1]). This may have been an adaptation to the extreme climatic and oceanic conditions (increases in temperature and oceanic anoxia; e.g. Kerr [89]) during this interval [67], linked to an inferred dramatic increase of CO_2_ in the atmosphere possibly triggered by enhanced global volcanism (e.g. the Ontong Java flood events). The high CO_2_ levels during this greenhouse period would also have led to increased oceanic acidity [90], which would have favored the ecological domination of the Textulariida, exemplified by the orbitolinids with their agglutinated tests, over those forms with biogenically precipitated calcitic tests that dominated before and after this period.

**Figure 12:**
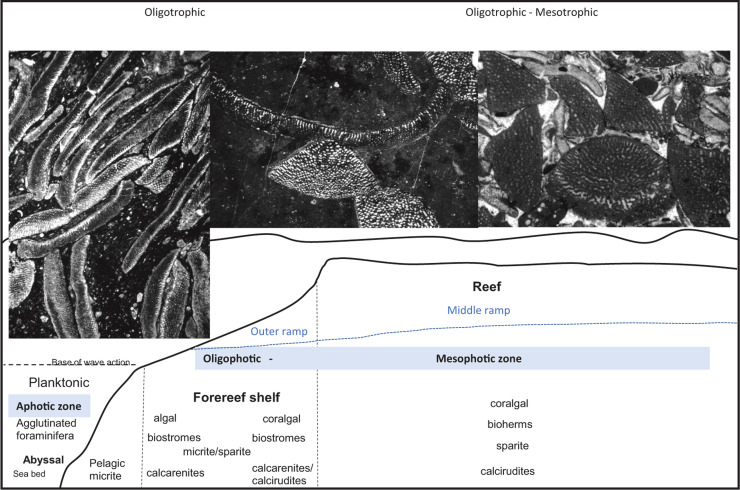
The facies range of the dominant orbitolinids in a Tethyan carbonate shelf. Integrated reef/ramp model for Cretaceous carbonates. The ramp model is indicated by the blue dotted line. In the case of gently sloping ramp, the outer ramp lithofacies are made of mudstones and wackestones, while in the middle ramp mudstone with carbonate nodules would develop. Orbitolinid photos are from the Langhan Formation, Tibet (see [31])

Evolving from earlier Valanginian forms, by the late Barremian (PZ Barremian 3), major new lineages of the agglutinated orbitolinids had appeared in Tethys (see [83]). These robust forms had the ability to survive in many shallow carbonate environments [91], however, they were most common in the outer platform ([1, 11, 67, 92]; and see Fig. 12).

As noted, we have shown that all cosmopolitan orbitolinids appeared in Tethys before migrating to other provinces. Likewise, we have seen that once established in the American and Western Pacific provinces, local provincial forms evolved, indicating that they were once again subsequently isolated from the Tethyan province. In previous studies of Cenozoic LBF, specifically the lepidocyclinids [93], the miogypsinids [94], the nummulitoids [95, 96] and the orthophragminids [97, 98], we have observed similar developments, with periods of migration from one province to another followed by subsequent isolation and development of local provincial linages. In these Cenozoic cases, the periods of inter-provincial migration coincided with major sea level regressions, while the subsequent provincial isolation coincided with global sea level transgressions. As observed in this study, it appears that a similar correlation occurs with the Cretaceous orbitolinids, with migrations from Tethys occurring during the time of Aptian sea level low stands (Fig. 6), followed by isolation when the sea level again rose in the Albian.

Thus, in Tethys, *P. lenticularis* (Plate 2, a; Fig. 10) first occurred in late Barremian (PZ Barremian 3, 127 Ma), 2 million years before its first appearance in the American and Western Pacific provinces (at the beginning of the Aptian, PZ Aptian 1, 125.0 Ma). This migration coincides with the global sea level regression that marks the beginning of PZ Aptian 1, and which culminates with the global sea level minimum at the end of PZ Aptian 2.

Similarly, the earliest *Mesorbitolina* (e.g. *Mesorbitolina lotzei*) appeared first in Tethys, in PZ Aptian 2, but are not recorded until PZ Aptian 3 in the Western Pacific province, and PZ Aptian 4 in the American province.

After the earliest migration in the Aptian, the American Province appears to have been again isolated from Tethys throughout the later Albian and the more advanced lineages of Group (v) (e.g. *Orbitolina, Conicorbitolina*) of the Tethyan provinces, which appeared in the late Albian, are not found in the Americas. The evolutionary patterns inferred from Tethyan species diverge from those observed in the Americas, confirming that these two provinces were isolated from each other at this time. The progressive changes seen in the different lineages are regarded here as examples of homoplasy, which resulted in the development of morphologically similar yet phylogenetically distinct forms with distinct biostratigraphic and paleogeographic characteristics.

The American orbitolinids became extinct at the end of the PZ Albian 2, 12.8 Ma earlier than those of Tethys (end Cenomanian 3). This event corresponds to the opening of the Western Interior Seaway triggered by sea level rises, and tectonic forces associated with the subduction of the Farallon Plate in the late Albian. This produced, for a period, an epicontinental sea over western North America that linked the tropical seas with a previously separate Artic Ocean. This fully open seaway persisted in the Albian and the Cenomanian, flooding the orbitolinids habitats with cooler deeper waters, and was probably the cause of the orbitolinids extinction in the American province.

In the Western Pacific province, the late Aptian to early Albian larger benthic foraminifera had their origin in Tethys. Following the early Albian migration of the Tethyan foraminifera, however, they seem to have become isolated in the South East Asian sub-province, again correlated with the early Albian sea level recovery. During the late Albian, the lineages evolved independently but in parallel to their Tethyan ancestors. The form *Conicorbitolina* sp. is similar to but different in specific characters from the Tethyan *C. conica* (d‘Archiac). This suggests that the migration of Albian foraminifera to the Western Pacific province was only possible for a limited period around the early Albian. Thereafter the orbitolinids of the South East Asian sub-province remained small, rare, and isolated from those in Tethys, as the exclusively Tethyan large species of *Orbitolina *never appeared in this sub-province. The orbitolinids do not survive the Albian-Cenomanian boundary, but unlike the Tethyan realm, disappeared completely from the Northwest Pacific subprovince at the end of PZ Albian 1 [32] and, from the South East Asian sub-province at the end of PZ Albian 4.

## Conclusion

Analysis of new material combined with a synthesis of the published literature has allowed the understanding of the global evolution and paleobiogeographic distribution of mid-Cretaceous orbitolinids within three LBF provinces; namely the Americas, Tethys, and the newly identified Western Pacific province.

We conclude that, unlike previously studied Cenozoic LBF forms, such as the lepidocyclinids [93], the miogypsinids [94], the nummulitoids [95] and the orthophragminids [96], which evolved first in the Americas and then migrated eastward to Tethys, the Mesozoic orbitolinids originated in the warm tropical shallow platforms of Tethys in the Early Cretaceous, Valanginian (PZ Valanginian 1). The subsequent paleogeographic migration during the global sea level low stands of the Aptian of members from orbitolinid Group (ii) and Group (v) was bidirectional, moving from Tethys westward to the Americas, and also eastward into the Western Pacific region. There is no evidence of a West to East trans-Atlantic migration, nor of migration of Western Pacific forms to Tethys.

We infer that migration stopped after rising sea level in the Albian. As species became geographically isolated, colonising new but ecologically similar habitats, they thrived and evolved similar but distinct parallel lineages, taking advantages of empty niches and optimum conditions. This example of parallel speciation is discussed by Schluter et al. [99], and probably reflects that all species shared a genetic predisposition to develop mutations of a specific, advantageous type, inherited from their last common ancestor.

The new understanding of the phylogenetic evolution of the Tethyan, Western Pacific and American orbitolinids presented in this paper, when combined with the improved understanding of their biostratigraphic ranges and facies relationships, provides the first global-scale understanding of their development, and so enhances their usefulness as a tool for the study of Early to mid-Cretaceous warm-water carbonate platforms, which are so important in today's hydrocarbon exploration.

## Data Availability

No further data was used in addition to referenced works.

## References

[1] BouDagher-Fadel MK (2018a). Evolution and geological significance of larger Benthic Foraminifera.

[2] BouDagher-Fadel MK (2018b). Revised diagnostic first and last occurrences of Mesozoic and Cenozoic planktonic foraminifera.

[3] Gradstein FM, Ogg JG, Schmitz MD, Ogg GM (2012). The geologic time scale.

[4] Husinec A (2001). *Palorbitolina lenticularis* from the northern Adriatic region: palaeogeographical and evolutionary implications. J Foraminiferal Res.

[5] Simmons MD, Williams CL, Hart MB (1992). Sea-level changes across the Albian-Cenomanian boundary in South-West England. Proc Ussher Soc.

[6] Simmons MD, Whittaker JE, Jones RW, Hart MB, Smart CW (2000). Proceedings of the Fifth International Workshop on Agglutinated Foraminifera.

[7] Carter DJ, Hart MB (1977). Aspects of mid-Cretaceous stratigraphic micropalaeontology. Bull British Museum (Natural History) Geol.

[8] Hart MB, Manley EC, Weaver PPE (1979). A biometric analysis of an Orbitolina fauna from the Cretaceous succession at Wolborough, S. Devon. Proc Ussher Soc.

[9] Hart MB, Durrance EM, Lamming DJC (1982). The geology of Devon.

[10] Hart MB, Williams CL (1990). The Upper Greensand in East Devon: new data but old problems. Proc Ussher Soc.

[11] Vilas L, Masse J-P, Arias C (1995). *Orbitolina* episodes in carbonate platform evolution, the early Aptian model from SE Spain. Palaeogeogr Palaeoclimatol Palaeoecol.

[12] Caus E, Teixell A, Bernaus JM (1997). Depositional model of a Cenomanian-Turonian extensional basin (Sopeira Basin, NE Spain): interplay between tectonics. Eustasy and biological productivity. Palaeogeogr Palaeoclimatol Palaeoecol.

[13] Clavel B, Charollais J, Busnardo R, Granier B, Conrad M, Desjacques P (2014). La plate-forme carbonatée urgonienne (Hauterivien supérieur-Aptien inférieur) dans le Sud-Est de la France et en Suisse: synthèse. Archiv Sci.

[14] Cherchi A, De Castro P, Schroeder R (1978). Sull’età dei livelli a Orbitolinidi della Campania e delle Murge Baresi (Italia meridionale). Boll Soc Nat Napoli.

[15] Saint-Marc P (1970). Contribution à la connaissance du Crétacé basal au Liban. Rev Micropaléontol.

[16] Cherchi A, Schroeder R, Bin Ghoth M (1998). Early Aptian orbitolinid foraminifera from the Qishn Formation of Al Mukalla (Hadramawt, Southern Yemen). Comparisons with adjacent regions. Z Geol Wissensch Berlin.

[17] Simmons MD, Hart MB, Hart MB (1987). Micropalaeontology of carbonate environments.

[18] Simmons MD, Simmons MD (1994). Micropalaeontology and hydrocarbon exploration in the Middle East.

[19] Masse J-P, Chartrousse A, Borgomano J (1998). The Lower Cretaceous (Upper Barremian-Lower Aptian) Caprinid Rudists from northern Oman. Géobios, mémoire spécial.

[20] Hughes GW, Powell AJ, Riding JB (2005). Recent Development in Applied Biostratigraphy. Micropalaeontol Soc Spec Publ.

[21] Cantrell DL, Griffiths CM, Hughes GW, Agar SM, Geiger S (2017). *Fundamental controls on fluid flow in carbonates: current workflows to emerging* technologies.

[22] Vahrenkamp VC (1996). Chemostratigraphy on the Lower Cretaceous Shu’aiba formation: a delta13^G^ reference profile for the Aptian stage from the southern Neo-Tethys Ocean. Am Assoc Petrol Geol Bull.

[23] Mehrnusch M (1973). Eine Orbitoliniden-Fauna aus der Unterkreide von Esfahan (Zentral Iran). Neues Jahrbuch fur Geologie und Palaontologie Monatshefte, Stuttgart.

[24] Shakib SS, Simmons MD (1994). Micropalaeontology and hydrocarbon exploration in the Middle East.

[25] Roozbahani PR (2011). Lithostratigraphy and biostratigraphy of the Lower Cretaceous of the Jalmajird area (northeast of Khomeyn, Central Iran Basin), Iran. GeoAlp.

[26] Schlagintweit F, Wilmsen M (2014). Orbitolinid biostratigraphy of the Top Taft Formation (Lower Cretaceous of the Yazd Block, Central Iran). Cretaceous Res.

[27] Rahiminejad AH, Hassani MJ (2016). Depositional environment of the Upper Cretaceous orbitolinid-rich microfacies in the Kuh-e Mazar anticline (Kerman Province, Central Iran). Historic Biol.

[28] Schroeder R (1975). General evolutionary trends in orbitolinas. Revista Española de Micropaleontología. Numero especial.

[29] Zhang B (1982). *Orbitolina* (foraminifera) from Xisang. Series of the Scientific Expedition to the Qinghai-Xisang Plateau. Palaeontol Xisang.

[30] Zhang B (1986). Early Cretaceous orbitolinids from Xainza and Baingoin, Xisang: Bulletin of the Nanjing Institute of Geology and Palaeontology. Acad Sin.

[31] BouDagher-Fadel MK, Hu X, Price GD, Sun G, Wang J-G, An W (2017). Foraminiferal biostratigraphy and palaeoenvironmental analysis of the mid-Cretaceous limestones in the southern Tibetan plateau. J Foraminiferal Res.

[32] Iba Y, Sano SI, Miura T (2011). Orbitolinid foraminifers in the Northwest Pacific: their taxonomy and stratigraphy. Micropaleontology.

[33] Yabe H, Hanzawa S (1926). Geological age of *Orbitolina* bearing rocks of Japan. Sci. Rep Tohoku Imperial Univ Second Ser.

[34] Hofker J (1963). Studies on the genus *Orbitolina* (Foraminiferida). Leidse Geol Medelin.

[35] Ujiié H, Kusukawa T (1968). *Orbitolina* (Cretaceous Foraminifera) from the Miyako Group, Iwate Prefecture, Northeast Japan. Mem Natl Sci Museum Tokyo.

[36] Matsumaru K (1971). Certain larger foraminifera from Japan. J Saitama Univ Fac Educ.

[37] Matsumaru K (1973). Atlas of Japanese Fossils Editorial Committee. Atlas of Japanese Fossils.

[38] Matsumaru K (2005). *Praeorbitolinoides*, a new Orbitolinid foraminiferal genus from the Lower Aptian (Cretaceous) of Hokkaido, Japan. Micropaleontology.

[39] Matsumaru K, Sudo K, Senaha T (1976). A discovery of *Orbitolina* from the calcareous sandstone of the Koma River, Hidaka-cho, Iruma-Gun, Saitama Prefecture, Japan. J Geol Soc Jpn.

[40] Salnikov BA, Tikhomolov YI, Poyarkova ZN (1987). Reference section of Cretaceous deposits in Sakhalin.

[41] Iba Y, Taki S, Yoshida K, Hikida Y (2005). *Orbitolina* bearing limestone pebbles from the lowermost part of Lower Yezo Group (Lower Cretaceous) in the Nakagawa area, northern Hokkaido, Japan and its significance. J Geol Soc Jpn.

[42] Matsumaru K, Furusawa A (2005). The present condition and subject of the Cretaceous Orbitolinid foraminiferal studies of Japan. J Saitama Univ Fac Educ.

[43] Matsumaru K, Furusawa A (2007). On Orbitolinid foraminifera from the Lower Aptian (Cretaceous) of Hokkaido, Japan. J Palaeontol Soc.

[44] Iba Y, Sano S (2006). *Mesorbitolina* (Cretaceous larger foraminifera) from the Yezo Group in Hokkaido, Japan and its stratigraphic and paleobiogeographic significance. Proc Jpn Acad Ser B.

[45] Bosellini A, Russo A, Schroeder R (1999). Stratigraphic evidence for an Early Aptian sea-level fluctuation: the Graua Limestone of south-eastern Ethiopia. Cretaceous Res.

[46] Luger P, Hendriks F, Arush M, Bussmann M, Kallenbach H, Mette W (1990). The Jurassic and Cretaceous of northern Somalia: preliminary results of the sedimentologic and stratigraphic investigations. Berliner geowissenschaftliche Abhandlungen (A).

[47] Bosellini A (1992). The continental margins of Somalia. Structural evolution and sequence stratigraphy. Am Assoc Petrol Geol Memoir.

[48] Cherchi A, Schroeder R (1999). Late Barremian orbitolinid Foraminifera from northern Somalia. Boll Soc Paleontol Ital Modena.

[49] Peybernès B, Blondeau A (1982). Colloque africain de micropaléontologie; IV, Notes consacrées a l’Afrique Occidentale et Equatoriale.

[50] Sen Gupta BK, Grant AC (1971). *Orbitolina*, a Cretaceous larger foraminifer, from Flemish Cap: paleoceanographic implications. Science.

[51] Schroeder R, Cherchi A, Tucholke BE, Vogt PR (1979). Initial reports of the Deep Sea Drilling Project.

[52] Douglass RC (1960). The Foraminiferal Genus *Orbitolina* in North America. Geol Surv Prof Paper.

[53] Meza J (1980). El género *Orbitolina* en México y su distribución estratigráfica. Revista delInstituto Mexicano del Petroleo.

[54] Pantoja-Alor J, Schroeder R, Cherchi A, Alencaster G, Pons JM (1994). Fossil assemblages, mainly foraminifers and rudists, from the early Aptian of southwestern Mexico. Paleobiogeographical consequences for the Caribbean Region. Rev Esp Paleontol.

[55] Omaña L, Alencáster G (2009). Lower Aptian shallow-water benthic foraminiferal assemblage from the Chilacachapa range in the Guerrero-Morelos Platform, south Mexico. Rev Mex Cienc Geol.

[56] Görög A, Arnaud Vanneau A (1996). Lower Cretaceous Orbitolinas from Venezuela. Micropaleontology.

[57] Schroeder R, Van Buchem FSP, Cherchi A, Baghban D, Vincent B, Immenhauser A (2010). Revised orbitolinid biostratigraphic zonation for the Barremian - Aptian of the eastern Arabian Plate and implications for regional stratigraphic correlations. GeoArabia Spec Publ Gulf PetroLink, Bahrain.

[58] Pan GT, Ding J, Yao DS, Wang LQ (2004). Guide book of 1:1,500,000 geologic map of the Qinghai-Xizang (Tibet) plateau and adjacent areas.

[59] Kapp P, DeCelles PG, Leier AL, Fabijanic JM, He S, Pullen A (2007). The Gangdese retroarc thrust belt revealed: Geological Society of America. GSA Today.

[60] An W, Hu X, Garzanti E, BouDagher-Fadel MK, Wang J, Sun G (2014). Xigaze forearc basin revisited (South Tibet): provenance changes and origin of the Xigaze Ophiolite. Geol Soc Am Bull.

[61] Witts D (2011). Stratigraphy and Sediment Provenance: The Barito Basin, Southeast Kalimantan, Indonesia, Unpublished PhD thesis.

[62] Sun G, Hu X, Sinclair HD, BouDagher-Fadel MK, Wang J (2015). Late Cretaceous evolution of the Coqen Basin (Lhasa terrane) and implications for early topographic growth on the Tibetan Plateau. Geol Soc Am Bull.

[63] Xu Y, Hu X, BouDagher-Fadel M, Sun G, Lai W, Li J (2019). The late Albian major transgressive event recorded in the epicontinental Langshan Formation in the central Tibet. Geol Soc Spec Publ.

[64] Bambach RK (2006). Phaenerozoic Biodiversity Mass Extinctions. The Annual Review of Earth and Planetary Science.

[65] Miller K, Kominz M, Browning J, Wright J, Mountain G, Katz M (2005). The Phanerozoic record of global sea-level change. Science.

[66] Hottinger L, Hedley RH, Adams CG (1978). Foraminifera 3.

[67] BouDagher-Fadel MK (2008). Developments in palaeontology and stratigraphy.

[68] Henson FRS (1948). Larger Imperforate Foraminifera of SouthWestern Asia (Families Lituolidae, Orbitolinidae and Meandropsinidae).

[69] Cherchi A, Schroeder R (1980). *Palorbitolinoides hedini* n. gen. n. sp., grand Foraminifère du Crétacé inférieur du Tibet méridional. Comptes rendus hebdomadaires des seances de l’Académie des sciences (Série D ,Sciences naturelles).

[70] Hashimoto W, Aliate E, Aoki N, Balce G, Ishibashi T, Kitamura N, Kobayashi T, Toriyama R (1975). Geology and Palaeontology of Southeast Asia.

[71] Hashimoto W, Matsumaru K (1977). *Orbitolina* from West Sarawak, east Malaysia. Geol Palaeontol Southeast Asia.

[72] Hashimoto W, Matsumaru K, Kobayashi T, Toriyama R (1974). Geology and Palaeontology of Southeast Asia.

[73] Yabe H (1946). On some fossils from the Saling Limestone of the Goemai Mts., Palembang, Sumatra. Proc Jpn Acad.

[74] Musper KAFR (1937). Geological map of Sumatra. Explanatory notes to sheet 16 (Lahat), scale 1:200 000. Dienst Mijnbouw Nederlandsch-Indie. Geological Survey of Indonesia.

[75] Harloff CEA (1929). Voorloopige mededeeling over de geologic van het Praetertiair van Loh Oelo in Midden-Java. De Mijningenieur.

[76] Brouwer HA (1934). Geologische onderzoekingen op het eiland Celebes. Verhandelingen van het geologisch mijnbouwkundig Genootschap voor Nederland en Koloniën. Geologische Serie, Deel.

[77] White LT, Hall R, Armstrong RA, Barber AJ, BouDagher-Fadel MK, Baxter A (2017). The geological history of the Latimojong region of western Sulawesi. J Asian Earth Sci.

[78] von Fritsch K (1879). Patellinen von der Westseite von Borneo. Palaeontographica.

[79] Sikumbang N (1986). Geology and tectonics of pre-Tertiary rocks in the Meratus Mountains, South-East Kalimantan, Indonesia. Ph.D. Thesis.

[80] Van Gorsel H (2014). An introduction to Mesozoic faunas and floras of Indonesia. Berita Sedimentol.

[81] Clavel B, Decrouez D, Charollais J, Busnardo R (2010). *“Paracoskinolina” praereicheli* n. sp., un orbitolinidé (Foraminifère) nouveau de l’Hauterivien supérieur et du Barrémien inférieur (Crétacé) á faciès urgonien (SE France, Jura franco-suisse, Préalpes suisses). Arch Sci.

[82] Maync W (1955). *Dictyoconus walnutensis* (Carsey, 1926) in the mid-Albian Guacharo limestone of eastern Venezuela. Contrib Cushman Found Foramin Res.

[83] Cherchi A, Schroeder R (2004). Evolution of orbitolinid foraminifers and anoxic events: a comment on an article by J. Guex. Eclog Geolog Helvet.

[84] Monreal R, Longoria JF, Bartolini C, Wilson JL, Lawton TF (1999). Geological Society of America Special Paper.

[85] Cherchi A, Schroeder R (2009). Revision of the orbitolinid foraminiferal genus Praeorbitolinoides MATSUMARU 2005 from the Aptian of Hokkaido, Japan. Micropaleontology, New York.

[86] Royer DL, Berner RA, Park J (2007). Climate sensitivity constrained by CO_2_ concentrations over the past 420 million years. Nature.

[87] Skelton PW, Masse J-P (2000). Synoptic guide to Lower Cretaceous rudist bivalves of Arabia. Soc Econ Paleontolog Mineral Spec Publ.

[88] Littler K, Robinson SA, Bown PR, Nederbragt AJ, Pancost RD (2011). High sea-surface temperatures during the Early Cretaceous Epoch. Nat Geosci.

[89] Kerr RA (2006). Creatures great and small are stirring the ocean. Science.

[90] Naafs BDA, Castro JM, De Gea GA, Quijano ML, Schmidt DN, Pancost RD (2016). Gradual and sustained carbon dioxide release during Aptian Oceanic Anoxic Event 1a. Nat Geosci.

[91] Arnaud Vanneau A (1980). Micropaléontologie, paléoécologie et sédimentologie d’une plateforme carbonatée de la marge passive de la Téthys: l’Urgonien du Vercors septentrional et de la Chartreuse (Alpes occidentales). Géol Alpine Mémoir.

[92] Pittet B, Van Buchem FSP, Hillgärtner H, Razin P, Grötsch J, Droste H (2002). Ecological succession, palaeoenvironmental change, and depositional sequences of Barremian-Aptian shallow-water carbonates in northern Oman. Sedimentology.

[93] BouDagher-Fadel MK, Price GD (2010). Evolution and paleogeographic distribution of the lepidocyclinids. J Foramin Res.

[94] BouDagher-Fadel MK, Price GD (2013). The phylogenetic and palaeogeographic evolution of the miogypsinid larger benthic foraminifera. J Geol Soc.

[95] BouDagher-Fadel MK, Price GD (2014). The phylogenetic and palaeogeographic evolution of the nummulitoid larger benthic foraminifera. Micropaleontology.

[96] Benedetti A, Less G, Parente M, Pignatti J, Cahuzac B, Torres-Silva AI (2018). *Heterostegina matteuccii* sp. nov. (Foraminiferida: Nummulitidae) from the lower Oligocene of Sicily and Aquitaine: a possible transatlantic immigrant. J Syst Palaeontol.

[97] BouDagher-Fadel MK, Price, GD (2017). The paleogeographic evolution of the orthophragminids of the Paleogene. J Foraminiferal Res.

[98] Özcan E, Mitchell SF, Less G, Robinson E, Bryan JR, Pignatti J (2019). A revised suprageneric classification of American orthophragminids with emphasis on late Paleocene representatives from Jamaica and Alabama. J Syst Palaeontol.

[99] Schluter D, Clifford EA, Nemethy M, McKinnon JS (2004). Parallel evolution and inheritance of quantitative traits. Am Naturalist.

[100] Arnaud Vanneau A (1975). Réflexion sur le mode de vie de certains Orbitolinidés (Foraminiféres) barrémo-aptiens de l’Urgonien du Vercors. Compte rendu des séances de la Société de physique et d’histoire naturelle de Genève.

[101] Banner FT, Simmons MD, Simmons MD (1994). Micropalaeontology and hydrocarbon exploration in the Middle East.

[102] BouDagher-Fadel MK, Wilson M (2000). A revision of some larger Foraminifera of the Miocene of South-East Kalimantan. Micropaleontology.

[103] Masse J-P (1976). Les calcaires urgoniens de Provence (Valanginian-Aptien), Stratigraphie, paléontologie, les paléoenvironnements et leur evolution.

[104] Walliser OH, Walliser OH (1995). Global Events and Event Stratigraphy.

[105] Witt W, Gökdağ, Simmons MD (1994). Micropalaeontology and Hydrocarbon Exploration in the Middle East.

